# Phishing vulnerability compounded by older age, apolipoprotein E e4 genotype, and lower cognition

**DOI:** 10.1093/pnasnexus/pgae296

**Published:** 2024-08-01

**Authors:** Didem Pehlivanoglu, Alayna Shoenfelt, Ziad Hakim, Amber Heemskerk, Jialong Zhen, Mario Mosqueda, Robert C Wilson, Matthew Huentelman, Matthew D Grilli, Gary Turner, R Nathan Spreng, Natalie C Ebner

**Affiliations:** Department of Psychology, University of Florida, 945 Center Dr, Gainesville, FL 32603, USA; Florida Institute for National Security, University of Florida, 601 Gale Lemerand Dr, Gainesville, FL 32611, USA; Department of Psychology, University of Florida, 945 Center Dr, Gainesville, FL 32603, USA; Department of Psychology, University of Florida, 945 Center Dr, Gainesville, FL 32603, USA; Department of Psychology, University of Florida, 945 Center Dr, Gainesville, FL 32603, USA; Department of Psychology, University of Florida, 945 Center Dr, Gainesville, FL 32603, USA; Translational Genomics Research Institute, 445 N 5th St 4th Floor, Phoenix, AZ 85004, USA; Department of Psychology, University of Arizona, 1503 E. University Blvd., Tucson, AZ 85721, USA; Translational Genomics Research Institute, 445 N 5th St 4th Floor, Phoenix, AZ 85004, USA; Department of Psychology, University of Arizona, 1503 E. University Blvd., Tucson, AZ 85721, USA; Department of Psychology, York University, 4700 Keele St, North York, ON M3J 1P3, Canada; Department of Neurology and Neurosurgery, McGill University, 1033 Pine Avenue West, Montreal, QC H3A 1A1, Canada; Department of Psychology, University of Florida, 945 Center Dr, Gainesville, FL 32603, USA; Florida Institute for National Security, University of Florida, 601 Gale Lemerand Dr, Gainesville, FL 32611, USA; Florida Institute for Cybersecurity Research, University of Florida, Malachowsky Hall, 1889 Museum Rd, Gainesville, FL 32603, USA; McKnight Brain Institute, University of Florida, 1149 Newell Dr, Gainesville, FL 32610, USA

**Keywords:** aging, fraud, phishing, APOE4, cognition

## Abstract

With technological advancements, financial exploitation tactics have expanded into the online realm. Older adults may be particularly susceptible to online scams due to age- and Alzheimer's disease-related changes in cognition. In this study, 182 adults ranging from 18 to 90 years underwent cognitive assessment, genotyping for apolipoprotein E e4 (APOE4), and completed the lab-based Short Phishing Email Suspicion Test (S-PEST) as well as the real-life PHishing Internet Task (PHIT). Across both paradigms, older age predicted heightened susceptibility to phishing, with this enhanced susceptibility pronounced among older APOE4 allele carriers with lower working memory. Additionally, performance in both phishing tasks was correlated in that reduced ability to discriminate between phishing and safe emails in S-PEST predicted greater phishing susceptibility in PHIT. The current study identifies older age, APOE4, and lower cognition as risk factors for phishing vulnerability and introduces S-PEST as an easy-to-administer, ecologically valid tool for assessing phishing susceptibility.

**Significance Statement**

As elements of daily life are increasingly online, there is a greater risk of financial exploitation perpetrated online. Older adults may be particularly vulnerable to online fraud due to age- and Alzheimer's disease (AD)-related declines in cognition. This work demonstrates that older age, genetic predisposition for AD, and lower working memory contribute to fraud and exploitation in cyberspace. These findings provide crucial insights into mechanisms of online deception risk toward informing public health efforts for reducing financial exploitation risk and optimizing prevention solutions among individuals at particular risk of neurodegenerative disease.

## Introduction

Financial fraud represents one of the most common forms of elder maltreatment (1–6). While people from any age group can be targeted by scammers, losses from fraud are not uniform across age groups, with greater monetary losses experienced by older adults (7). According to the Federal Bureau of Investigation (FBI), in 2021 there were 92,371 older victims of fraud resulting in $1.7 billion in losses, which was a 74% increase in losses compared to 2020 (8). Financial losses due to exploitation can have devastating effects on health and independence of older adults (9–13).

Despite accumulation of world knowledge and life experience with age, older adults significantly decline in fluid cognition, i.e. the ability to process and integrate information and solve problems (14), resulting in reduced decision-making capacity (15, 16) and greater susceptibility to deception (12, 17). For example, declines in episodic memory, processing speed, and working memory were associated with greater self-reported scam susceptibility among older adults (18). Similarly, a recent study reported that reduced conscious deliberation measured via executive functioning ability was associated with lower deception detection in older adults, with the strongest associations observed in individuals 80 years and over (19). In addition, reduced sensitivity to negative arousal cues likely underlies poorer deception detection with age. For example, relative to young adults, older adults showed diminished activity in the anterior insula and caudate when anticipating monetary losses (vs. gains) (20) and were more trusting to negative cues of trustworthiness such as untrustworthy faces (21–23) and fake news (24). Furthermore, age-related increased vulnerability to deception is also associated with neurobiological changes with age (12, 25, 26). For example, relative to age-matched controls who avoided exploitation, financially exploited older adults showed cortical thinning in the anterior insula, a brain region implicated in integrating emotionally valenced internal and external stimuli (27). Exploited older adults also showed decreased functional coupling within the default network and increased functional coupling between brain networks (26), two hallmark patterns of age-related brain changes (28).

Alzheimer's disease (Ad) further exacerbates the risk of financial exploitation in aging. Cross-sectional and longitudinal evidence supports that relative to age-matched controls, older adults with mild cognitive impairment (MCI) and Ad experience declines in financial capacity (29, 30), lower scam awareness (31), and greater self-reported scam susceptibility (32). Furthermore, declines in fluid cognition, reduced volume in brain structure related to Ad pathology (e.g. medial prefrontal cortex, lateral parietal regions), and greater β-amyloid burden contributed to scam susceptibility in older individuals with MCI and Ad (29–32). However, to date, less is known about susceptibility to scams among generally healthy older adults at risk of developing Ad, despite evidence that Ad risk factors impact cognition and brain aging in the absence of overt Ad symptoms (33, 34). In particular, presence of the apolipoprotein E e4 (APOE4) allele is a robust Ad risk factor (35, 36) that can be studied more readily than other risk factors (e.g. amyloid and/or tau pathology) and is linked to poorer cognition as well as pathological brain changes (37–39). For example, the presence of the APOE4 risk allele has been associated with reduced volume in the medial temporal lobe (40), a region associated with scam vulnerability among older adults (26, 32). Therefore, being a carrier of APOE4 may constitute an Ad risk factor associated with greater deception vulnerability, even before emergence of the clinical syndrome.

The rapid shift to a digital world confronts the aging individual with drastically new contexts and risks (12). Email phishing, for example, has become a popular deception tool with immense costs to the individual and society. According to the FBI, phishing was among the most highly reported internet scams, with 300,497 victims reporting over $52 million in losses (8). Importantly, phishing emails are among the most common methods of contact used by fraudsters targeting older adults (7). While older adults (65 years and over) constitute only about 16.8% of the US population (41), they often hold positions of power in organizations and politics, have retirement savings accumulated over the course of their life, and make important financial and health-related decisions. Therefore, online deception via phishing emails of these individuals can result in negative consequences with broad societal impact and there is an urgent need to identify risk factors underlying phishing email detection.

Due to growing risks online and associated costs of online deception in aging, there has been an increased attention on the investigation of age-related changes in susceptibility to phishing emails. To this end, previous studies have conducted naturalistic field experiments, in which phishing susceptibility was measured by sending simulated phishing emails to participants without their knowledge, and consistently reported an age-related increase in vulnerability to phishing emails (17, 42, 43). Other studies which measured phishing detection performance by focusing on lab-based assessments under different task contexts, however, reported more mixed findings regarding age effects. For instance, one study asked participants to rate the suspiciousness of phishing and safe emails and found reduced discrimination ability between phishing and safe emails with increasing age (44). In contrast, Sarno et al. (45) required participants to classify emails as “legitimate” or “phish” and reported greater detection of phishing emails with age. Similarly, a study which had participants browse safe and phishing websites to see whether or not they divulge sensitive information found that young adults were more vulnerable to phishing than older adults (46). To resolve this mixed pattern of findings across paradigms and contexts, there is a critical need for unifying lab-based assessment with assessment of actual behavior “in the wild” toward the development of ecologically valid measures of phishing susceptibility in aging. Further, while there is emerging evidence that declines in memory functioning may drive age-related increase in susceptibility to email phishing (17), current knowledge regarding factors that contribute to age effects in phishing email detection is still very limited.

**Fig. 1.**
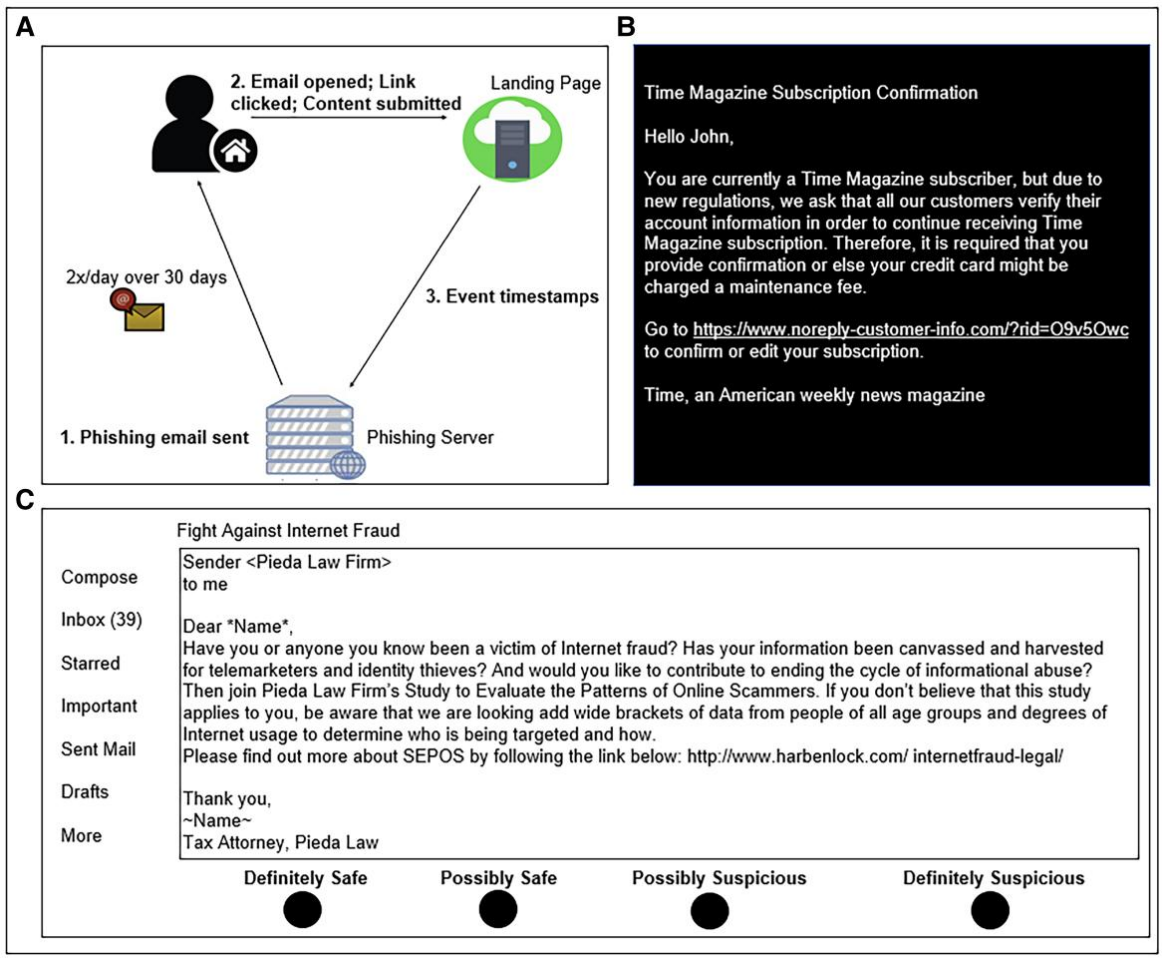
Phishing email detection paradigms. A) PHIT: unbeknownst to them, participants received 60 simulated phishing emails over 30 days (two emails per day) in their personal email inbox and the PHIT infrastructure recorded participants’ interactions with these emails (i.e. number of emails opened, number of email links clicked, and whether a participant submitted content on the landing page). B) Sample of phishing email in PHIT, which each was personalized (using participant's first name). C) S-PEST: schematic of the display seen by participants to rate each of 40 emails (20 phishing and 20 safe, presented one at a time, in randomized order) on suspiciousness using a four-point scale from “definitely safe” to “definitely suspicious.” The email displayed is a phishing email.

As part of a larger project on aging and deception (see also Heemskerk et al. (47)), the present study leveraged the newly developed PHishing Internet Task (PHIT; Figure 1A; adapted from Lin et al. (43) and Oliveira et al. (48)) to assess behavior-based real-life susceptibility to phishing. This task was conducted out of the participants’ homes where they received simulated phishing emails unbeknownst to them (Figure 1B for sample email) over a 30-day period (two emails per day). Our infrastructure recorded whether participants opened the emails, clicked on the links embedded in the emails, and submitted any information on the landing pages that accompanied the emails. Participants also completed the short version of the Phishing Email Suspicion Test (S-PEST; adapted from Hakim et al. (49) and Grilli et al. (44)), a lab-based phishing task that contains 40 emails (20 safe and 20 phishing). In this task, participants are asked to rate each email on its suspiciousness using a four-point scale that ranges from “definitely safe” to “definitely suspicious” (Figure 1C). Furthermore, we assessed each participants’ cognitive functioning using a test battery which involved measures of semantic and episodic memory, working memory, speed of processing, verbal fluency, reasoning, and task switching (50–52). Ad risk status was determined based on genotyping for APOE4 using self-collected dried blood samples (see Materials and methods for details of procedures).

Our study investigated whether and how age, Ad genetic risk, and cognitive status contributed to increased susceptibility to email phishing. Critically, we used both lab-based and real-life phishing tasks toward the validation of a novel, easy-to-administer paradigm (S-PEST) with excellent potential for translation to clinical settings. We hypothesized that phishing email detection would decline with older age, both in the lab and in real life; older age, APOE4-positive status, and lower cognitive functioning would compound phishing susceptibility; and these findings would replicate from the lab to real-life phishing contexts.

## Results

### Participants

The sample for this analysis comprised 182 adults from a wide age range (18–90 years). Table 1 presents sample demographics. All participants were in good health, with no history of an unstable medical illness (e.g. metastatic cancer) or primary degenerative neurological disorders (e.g. traumatic brain injury, Ad). The Telephone Interview for Cognitive Status (TICS; Brandt et al., 1988) was used to screen for baseline cognitive functioning among individuals over 55 years and all participants had normal cognitive functioning (TICS score range = 29–41, *M* = 35.5, *SD* = 2.54). The sample comprised 46 individuals (25%; *M*_age_ = 42 years; 82% female) with at least one copy of the APOE4 allele (i.e. ε2/ε4, ε3/ε4, or ε4/ε4*)* and 136 individuals (75%; *M*_age_ = 48 years; 77% female) without an APOE4 allele (i.e. ε2/ε2, ε2/ε3, or ε3/ε3). This distribution aligns with previous reports (53, 54).

**Table 1.** Sample description.

	Participants (*N* = 182)
	Mean/% (SD)
**Age** (in years)	46.53 (22.62)
**Education** (in years)	15.8 (2.94)
**Sex** (female)	78.02%
**Race**	
White	75.96%
Black/African	8.46%
Asian	7.79%
Other	7.79%
**Married/in a relationship** (yes)	48.13%
**Living alone** (yes)	33.76%
**Employed** (yes)	42.14%
**Income**	
<$24,999	35.00%
Between $25,000 and $99,999	43.75%
>$100,000	21.25%
**Computer literacy score**	0.95 (0.09)
**APOE allelic frequency**	
ε2ε2	0.55%
ε2ε3	14.83%
ε2ε4	1.65%
ε3ε3	58.24%
ε3ε4	23.08%
ε4ε4	1.65%

Three participants had missing data on years of education; one participant on race; four participants on marital status; six participants on living condition; four participants on employment status; and three participants on income. Computer literacy was measured via a test of knowledge of symbols and terms related to computers and other electronic equipment (higher scores reflect greater computer literacy).

### Older age, APOE4-positive status, and lower cognitive functioning predicted worse phishing detection

We conducted separate regression models for S-PEST and PHIT, with chronological age, APOE4 status (APOE4 carriers vs. noncarriers), and cognition scores (i.e. semantic memory, episodic memory, working memory, speed of processing, verbal fluency, reasoning, and task switching) as predictors, while controlling for participant sex, years of education, income, marital status, and computer literacy to account for differences in computer knowledge. S-PEST was scored using standard signal detection theory to compute discrimination ability (44), with higher scores indicating a participant's greater ability to discriminate between phishing and safe emails. Susceptibility in PHIT was computed as the sum of the actions (i.e. opening, clicking, submitting information) taken at least once, with higher scores indicating a participant's greater susceptibility to phishing emails in real life. See Materials and methods for details on scoring and statistical modeling.

**Fig. 2.**
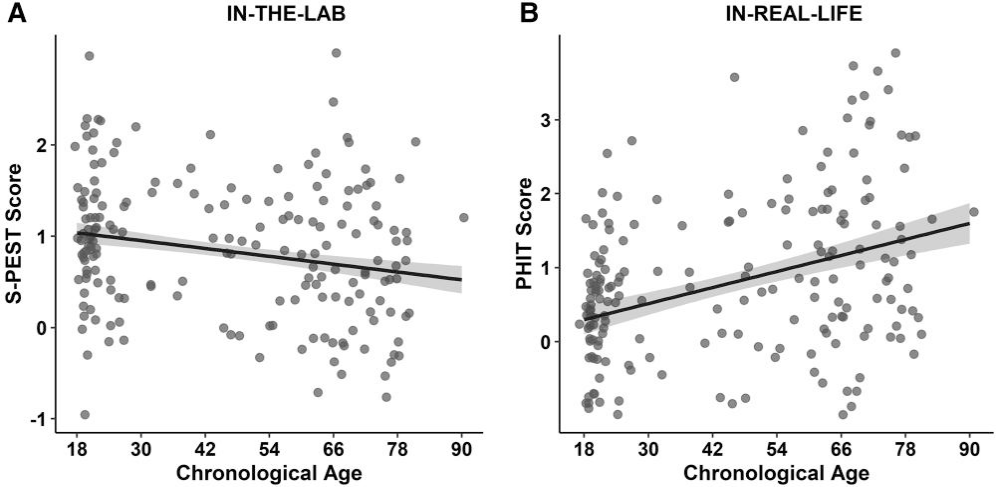
Older age impaired email phishing detection. Greater chronological age was associated with both A) lower discrimination between phishing and safe emails in S-PEST and B) greater susceptibility to phishing emails in PHIT. Each dot corresponds to a participant (jittered for visualization). Shaded areas around the regression lines reflect the 95% CI. Higher scores in S-PEST indicate greater lab-based discrimination ability between phishing and safe emails. Higher scores in PHIT indicate greater real-life email phishing susceptibility.

Our models revealed a main effect of *chronological age* on phishing detection performance both in the lab and in real life. In particular, the ability to discriminate between phishing and safe emails in S-PEST declined with age (*B* = −0.008, *t* = −3.83, *P* < 0.001, 95% CI = [−0.012, −0.004]; Figure 2A) and older age was associated with greater susceptibility to phishing emails in PHIT (*B* = 0.027, *z* = 3.12, *P* = 0.002, 95% CI = [0.010, 0.045]; Figure 2B).

**Fig. 3.**
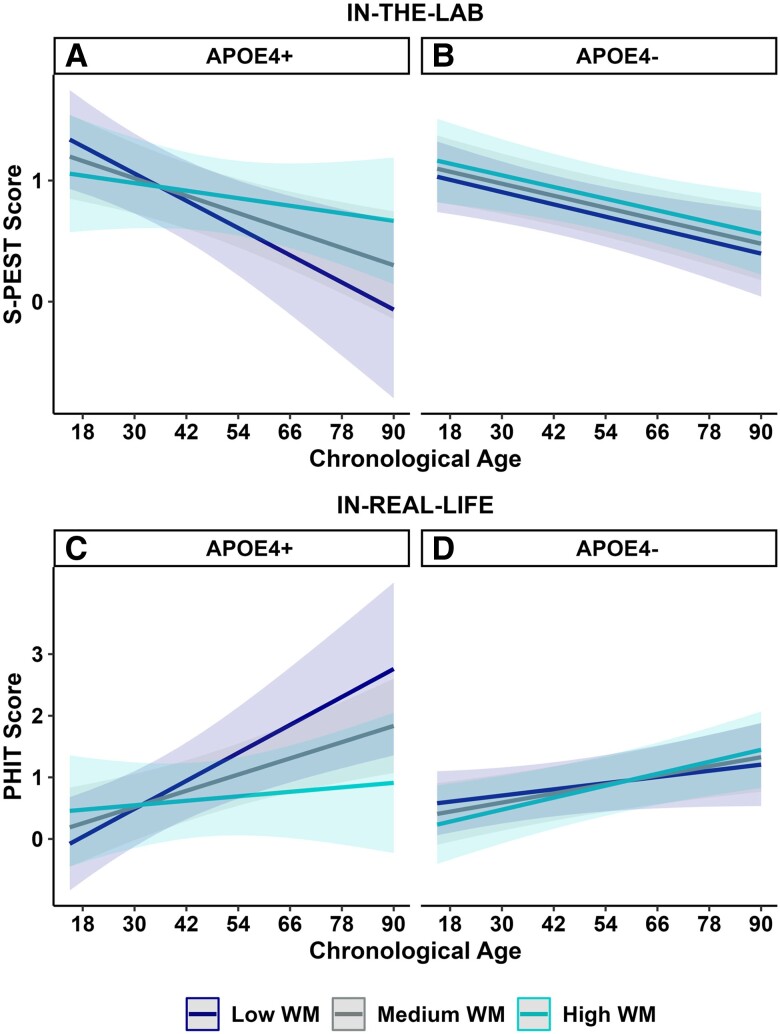
Older age, APOE4-positive status, and lower cognitive functioning were related to reduced email phishing detection. Older APOE4 carriers with lower working memory (WM) showed both A) lower discrimination between phishing and safe emails in S-PEST and C) greater susceptibility to phishing emails in PHIT. Age did not interact with cognitive status to predict phishing detection neither B) in the lab nor D) in real life among noncarriers of the APOE4 allele. Shaded areas around the regression lines reflect the 95%CI. Cognition scores were submitted as continuous variables in the analysis but are categorized for plotting purposes; medium WM indicates the mean residual WM score in the current sample while low and high levels indicate 1 SD below and above the mean residual WM score, respectively. Higher scores in S-PEST indicate greater lab-based discrimination ability between phishing and safe emails. Higher scores in PHIT indicate greater real-life email phishing susceptibility. APOE4±, apolipoprotein E e4 carriers/noncarriers.

Further, the interaction between *chronological age*, *APOE4 status*, and *cognitive functioning* was also significant (S-PEST: *B* = 0.008, *t* = 2.12, *P* = 0.036, 95% CI = [0.001, 0.016]; PHIT: *B* = −0.035, *z* = −2.03, *P* = 0.042, 95% CI = [−0.069, −0.001]). To interpret this significant three-way interaction, we compared the effects of age and cognitive functioning on S-PEST and PHIT separately for APOE4 carriers vs. noncarriers. For S-PEST, older age and lower working memory (measured via Digit Span Backwards; Tun and Lachman (51)) predicted reduced discrimination performance between phishing and safe emails in the lab, with this effect, however, only present among APOE4 carriers (*B* = 0.009, *t* = 2.34, *P* = 0.027, 95% CI = [0.001, 0.018]; Figure 3A) but not APOE4 noncarriers (*B* = −0.001, *t* = 0.22, *P* = 0.830, 95% CI = [−0.002, 0.003]; Figure 3B). A comparable effect was observed for PHIT in that older age and lower working memory (measured via Digit Span Backwards; Tun and Lachman (51)) predicted increased susceptibility to phishing emails in real life, with this effect again present among APOE4 carriers (*B* = 0.041, *z* = 2.05, *P* = 0.040, 95% CI = [0.002, 0.078]; Figure 3C) but not APOE4 noncarriers (*B* = 0.001, *z* = 0.13, *P* = 0.894, 95% CI = [−0.009, 0.010]; Figure 3D)^a^. No other effects were significant at *P* < 0.05.

### Reduced email phishing detection in the lab was related to increased email phishing susceptibility in real life

Performance in S-PEST and PHIT was significantly related (*r* (182) = −0.21, *P* = 0.006), suggesting that participants with lower discrimination ability between phishing and safe emails as measured in the lab-based S-PEST were more likely to fall for phishing emails in the real-life PHIT^b^.

## Discussion

Increased internet use has resulted in online deception tactics like email phishing to become a major public health concern, leading to dramatic financial (e.g. fraud and exploitation (7)) and psychological (e.g. depression and suicide (9, 11)) consequences, with particular risks among aging individuals (12, 13). While prior work has reported an age-related increase in vulnerability to phishing emails (17, 42–44), results are currently mixed with some studies providing evidence for greater phishing detection ability with age (45, 46, 55).

Our newly developed PHIT paradigm, which allowed us to obtain a real-life behavioral measure of phishing susceptibility, not only goes beyond previous self-report but also is placed in the everyday life of our participants, thus offering ecological validity by assessing participants’ susceptibility to email phishing as it occurs in naturalistic contexts. Further, our data revealed that this real-life measure of phishing susceptibility (assessed via PHIT) was correlated with phishing detection in the well-controlled lab context (assessed via S-PEST). Importantly, using for the first time the lab-based S-PEST in combination with the real-life PHIT, we observed an age-related decline in phishing email detection ability across both assessment contexts. Further, informing individualized risk profiles, our findings demonstrate that lower cognition combined with higher genetic risk of developing Ad contributes to greater phishing susceptibility in aging. In particular, reduced phishing detection was specifically pronounced among older individuals who were carriers of the APOE4 risk allele and with lower working memory. This finding aligns with previous research which suggests that decision making under risk and ambiguity tends to impair early in the progression of Ad, with this impairment of decision-making capacity exacerbated by deficits observed in basic cognitive functioning (56).

Interestingly, our results highlight working memory as the main construct influencing phishing vulnerability among older APOE4 carriers given that none of the other measures, which tapped into different cognitive processes (e.g. reasoning, processing speed, semantic and episodic memory), predicted phishing email detection. Working memory represents a series of operations such as active maintenance of goals and manipulation of task-relevant information (57, 58) that are domain general and common to other cognitive functions (59, 60). Importantly, working memory is among early cognitive impairments in healthy aging that reliably predict the progression from MCI to Ad even in the absence of deficits in episodic memory (61, 62). Thus, although speculative, it is possible that declines in working memory have a greater impact on phishing and other forms of deception detection among older adults who are in the early stages of Ad pathology, which APOE4 carriers are at higher risk to develop. Meta-analytical evidence demonstrated that cognitive training that targets working memory processes results in reliable improvements on the trained task as well as shows near- and far-transfer tasks (63). Thus, future intervention on reducing deception risk in aging could entail working memory training among older individuals who carry an APOE4 allele.

As touched on earlier, past measurement of phishing susceptibility involved diverse methodological approaches, ranging from lab-based assessments across task contexts (e.g. web browsing (46); roleplaying a person checking their emails (55); email classification (45)) to naturalistic field experiments (e.g. sending simulated phishing emails to participants’ email addresses (17, 42, 43). Going beyond this previous research, the current study established ecological validity of S-PEST as an in-lab measure by showing an association between S-PEST and PHIT performance, whereby people who perform poorly on S-PEST were more susceptible to real-life phishing. This finding complements our previous work in which we found that emails that were rated more suspicious in the lab were more likely to phish people in the real world using a separate group of participants (49). Moving forward, item response theory will allow refining S-PEST by identifying items that are most sensitive at detecting online deception and exploitation risk toward launching the application of S-PEST as a short, easy-to-administer diagnostic assessment tool in clinic and practice.

While the current study sheds light on risk profiles associated with vulnerability to email phishing, it is limited in scope of investigation. Future work should consider both breadth (coverage) and depth (specificity) of analysis to better delineate a diverse set of interindividual and contextual factors contributing to deception risk and design of interventions. For instance, to expand the breadth of investigation, future research could extend our investigation into socioemotional functioning domains by considering variables such as depression and social isolation among older adults can exacerbate deception risk (64, 65).

Also, the present study used the Brief Test of Adult Cognition by Telephone (BTACT) (51), as a brief measure to capture basic cognitive abilities (e.g. attention, working memory, fluency, episodic memory). In future extension of this work, a more extensive cognitive characterization of participants, including cognitive capacities such as complex problem solving and decision making, would be beneficial to delineate the role of specific cognitive capacities in phishing detection among older adults. Additionally, while our investigation did not specifically focus on financial exploitation, participants’ perceived financial status and income inequality (66–68) as well as objective measures of their financial status such as household income, household assets and debt (69) would be beneficial to assess to determine their impact on phishing susceptibility.

Of note, our approach is limited in that APOE4-positive status represents only one genetic indicator of Ad risk; future studies could benefit from obtaining genome-wide polygenic risk scores and additional biomarkers such as amyloid-β, tau protein levels, and blood-based biomarkers (e.g. ratio of amyloid-β peptides: Aβ42/Aβ40, levels of phosphorylated tau isoforms) to enhance the depth of investigation on cognitive disease-related risk profiles and deception. Lastly, the current study adopted a cross-sectional design with a primarily non-Hispanic White, well-educated, and largely female sample, and results will need to be confirmed via longitudinal or cross-sequential design including individuals from diverse backgrounds, to allow dissociation of age from cohort effects in a more representative adult lifespan sample for broader generalizability of findings.

Interestingly, and consistent with mounting evidence on increased variability in performance among older compared to younger adults (70–73), older age was associated with greater variation in phishing detection ability (see Figure 2). This pattern could be a result of greater age-related variation in socioemotional processes (e.g. theory of mind, loneliness (12, 74–76)) with relevance to phishing detection, which were not examined here; or could be due to increased age-related variation in brain structure and function in regions with particular relevance for deception detection (e.g. insula (12, 22, 26, 30, 77)) or in physiological response subserving deception detection (e.g. interoceptive awareness (47, 78)). Systematically determining such moderating variables in future research will further inform the design of interventions aimed at reducing exploitation risk among older adults (79). Also, future studies that specifically delineate cognitive, socioemotional, and brain-related characteristics of older adults who perform particularly poor or particularly well in phishing detection (e.g. by comparing super agers and poor agers (80)) will increase understanding of risk vs. protective profiles.

The present study takes a unique and important step toward a more naturalistic, real-life behavior-based approach at determining phishing susceptibility among adults of different ages, and it identifies crucial risk factors (age, genetic risk for Ad, cognitive status). Crucial extension of this work includes recruitment of community-dwelling older adults who are particularly vulnerable (e.g. have been, or continue to be, victims of fraud in real life (26)) as well as are from disadvantaged backgrounds, a population segment that is currently severely understudied regarding exploitation. Also, moving forward, prospective studies are needed to allow prediction of future fraud susceptibility based on lab-generated risk profiles. Integration of public records will be essential to start addressing challenges with underreporting of exploitation in real life (7, 81) and underestimated base rates. Machine learning approaches in behavioral analytics will further facilitate the profiling of consumer behavior and fraud risk (e.g. detection of irregularities in bank transaction trends for prediction of fraud risk (82)). Finally, knowledge transfer into the community and policy stakeholders (e.g. elder community care, law) will be essential.

## Conclusion

In conclusion, our work provides crucial insights into mechanisms of online deception risk toward informing public health efforts for reducing financial exploitation risk and optimizing prevention solutions among older adults. Results from this study importantly advance understanding of the role of older age, presence of the APOE4 allele, and lower working memory as risk factors that contribute to fraud and exploitation in cyberspace. Also, integrating in-lab and real-life measures of phishing susceptibility, our work provides a crucial first step in the development of easy-to-administer, ecologically valid assessment tools for those at particular risk of neurodegenerative disease. Finally, current training and warning solutions for online scams and threats operate under the implicit assumption that one-size-fits-all. However, our work suggests that this is not the case and that rather an individualized approach is warranted to assist the particularly vulnerable aging individual when navigating online.

## Materials and methods

### Study overview and recruitment

This paper reports findings from a larger project on susceptibility to deception in aging (see Supplemental Figure S1 for an overview of the larger project). All procedures were approved by the University of Florida's Institutional Review Board (IRB protocol #: IRB201801057), and all participants provided written informed consent. Participants were told that the study was about how well they “understand themselves and others.” They were informed that the study comprised sessions completed at home via zoom as well as lab visits both with an experimenter present and questionnaires sent to their personal email for completion on their own. Participants were not told that they would receive simulated phishing emails from our study team while enrolled in the study. This approach was used to prevent any changes in behavior if the real study purpose had been known.

Participants were recruited from the community in North Central Florida via university participant registries, senior citizen facilities, ResearchMatch, and word of mouth. Participants were eligible for the study if they were between 18 and 100 years old, able and willing to provide informed consent, English-speaking, on a stable regimen of medications, had at least an eighth grade education, and had access to a personal email account they used regularly.

As depicted in Supplemental Figure S1, data analyzed in the paper comprised the following components of the larger project: (i) a *screening session* which involved obtaining informed written consent, determining overall health and cognitive status (via TICS (83)), and collecting demographic information; (ii) a 30-day *at-home portion* during which participants completed three online questionnaire packages (from which the computer literacy scale, adapted from Sengpiel and Dittberner (84), was included in this paper) and received, unbeknownst to them, two simulated phishing emails each day to their personal email account as part of PHIT; and (iii) a *follow-up in-lab session* in which participants completed S-PEST, a series of cognitive functioning measures (50–52), and provided dried blood samples to determine APOE4 allele status. Participants were debriefed and compensated with up to $430 upon study completion. Upon completion of all study components, participants were compensated with up to $430 and debriefed regarding the real purpose of the study. They were then given the option to withdraw their data now that they had learned about the real study purpose. All participants granted permission to use their data.

### Measures

This paper analyzed data from (i) two phishing paradigms (PHIT and S-PEST) to determine email phishing detection ability, (ii) cognitive functioning measures, and (iii) dried blood sampling for APOE4 genotyping. Below, each of these measures is described in more detail.

#### Phishing email paradigms

##### PHishing Internet Task (PHIT)

PHIT (Figure 1A) comprised 120 simulated phishing emails created by our research team. Each email contained a subject line and was personalized by using the participant's first name. The body of each email comprised HTML elements and images related to the email content along with a link that directed participants to an accompanying landing page, also created by our research team and that contained fields requesting submission of information (e.g. email, phone number).

Each participant was sent a subset of 60 emails (two emails per day over 30 days; see Figure 1B for a sample email). Emails were pseudo-randomly sampled from a larger pool of emails following a counterbalancing scheme that ensured equal numbers of emails from impersonated vs. fictitious companies/entities and leveraging weapons of influence (Authority, Scarcity, Social Proof, Liking/Kindness, Reciprocity, Commitment) (85) to ensure a diverse set of emails. The first of each day's two emails was sent in the morning (at random between 8 AM and 11:55 AM) and the second around late afternoon (at random between 3 PM and 9:55 PM), with these times chosen to mimic typical work and leisure times.

The PHIT infrastructure was hosted on three Amazon EC2 virtual servers (i.e. instances; https://aws.amazon.com/ec2/), with one domain per server. Servers and domains were configured with standard IT protocols (e.g. SSH, SPF, DMARC) to secure participant data and prevent them from being used by malicious agents. Sender addresses were randomly determined to be impersonated (e.g. google@domain.com) or fictitious (e.g. marylou@domain.com) at the time of scheduling emails for send-out. Each server contained a different set of fictitious sender addresses to introduce variability to spam filters (e.g. in gmail, hotmail), and throughout the duration of the project, servers were periodically refreshed with new domains and new sender addresses to improve deliverability, mitigate spam filtering, and keep a good sending reputation. Email send-outs were scheduled separately for each participant via the open-source phishing framework Gophish (https://getgophish.com/) and sent through mail server IPs provided by the third-party service Mailgun (https://www.mailgun.com/). Responses were recorded separately for each participant via the Gophish listener, which used the SQLite database to store (i) email opens, (ii) email link clicks, and (iii) submission of information on the landing pages, which was captured via text entry data. Responses captured by the Gophish listener were coded based on whether the participant opened at least one email (0 = no; 1 = yes); clicked on at least one email (0 = no; 1 = yes); and submitted information on the landing page at least once (0 = no; 1 = yes). Susceptibility in PHIT was computed as the sum of these actions taken at least once by a participant and ranged from 0 to 3, with higher scores indicating a participant's greater susceptibility to phishing emails in real life (i.e. lower ability to detect phishing emails).

##### Short Phishing Email Suspicion Test (S-PEST)

S-PEST (Figure 1C) contained 40 emails sampled from the original Phishing Email Suspicion Test(49). To assure a diverse set, emails varied in legitimacy (safe vs. phishing), source (real vs. simulated), and whether a link was embedded in the email body or whether the email contained an attachment.

Participants received written task instructions and two practice trials. In particular, participants were informed that they would see a series of emails as in a regular email inbox, with some of these emails phishing and some safe messages. Participants were asked to categorize each email via keyboard press regarding the level of suspiciousness on a four-point scale from 1 = definitely safe to 4 = definitely suspicious. Email presentation order was randomized, and each email was presented for 120 seconds during which participants were instructed to give their response. At the end of the task, participants received an individualized score based on their task accuracy. S-PEST was coded in PsychoPy (86) and presented via Pavlovia (https://github.com/zmhakim/s-pest). The total duration of the task was about 10 minutes.

S-PEST was scored using standard signal detection theory to compute discrimination ability (i.e. *d*-prime denoted as *d′*). Phishing emails were considered as “signal present,” and correct responses of “definitely suspicious” or “possibly suspicious” for phishing emails reflected hits, whereas incorrect responses of “definitely safe” or “possibly safe” reflected misses. For safe emails, responses of “definitely safe” or “possibly safe” reflected correct rejections, whereas responses of “definitely suspicious” or “possibly suspicious” reflected false alarms. Using the formula *d′* = *z*(*H*)−*z*(*F*), *d′* was calculated for each participant across all emails, with higher *d′* indicating a participant's greater ability to discriminate between phishing and safe emails (i.e. greater ability to detect phishing emails).

#### Cognitive functioning measures

##### Automated Operation Span (OSPAN) task

The automated OSPAN (52) is a computerized version of the original OSPAN (87), measuring working memory capacity. The task requires participants to solve a series of math operations while trying to remember, in order, a series of unrelated letters. In particular, participants are first shown a simple math problem (e.g. (1 × 2) + 1 = ?). Participants click on the screen to move on as soon as they solve the problem. Next, a number appears on the screen (e.g. 3) and participants indicate whether the number represents the correct answer to the math problem. This is then followed by a single, unrelated letter (e.g. P) presented for 800 ms. After completing a block of trials (ranging from 3 to 7), participants are shown a 3 × 4 grid of letters and instructed to select the letters they have seen before, in the order they were presented, followed by feedback regarding their performance (correct math problems solved as well as correct letters recalled) for 2,000 ms before the next block starts. The automated OSPAN has both good internal consistency (alpha = 0.78) and test–retest reliability (*r* = 0.83) and takes approximately 20–25 minutes. For analysis, we used the absolute automated OSPAN score, reflecting the sum of all trials in which all letters were recalled in the correct serial order.

##### Brief Test of Adult Cognition by Telephone (BTACT)

The BTACT (51) contains seven subscales that assess key aspects of cognition. *Episodic memory* is measured with Word List Immediate Recall and Word List Delayed Recall subscales, which involve immediate recall and delayed retrieval of a list of 15 words. The memory composite score reflects the average of *z*-scores for the two subscales standardized to *z*-score (DeBlasio et al. 2021). *Working memory* is measured with the Digit Span Backwards subscale in which strings of numbers are repeated in reverse order and the length of the strings of numbers increased with each correct repetition (ranging from 2 to 8 digits). The Backward Digit Span is scored from 0 to 8 corresponding to the longest set of digits correctly repeated backwards. *Verbal fluency* is measured by the Category Fluency subscale in which participants list as many items as they could remember belonging to a particular category (i.e. “animals”) in 60 seconds. The score reflects the total number of unique animals listed. *Task-switching* ability is measured by the Stop and Go Switch Task. On No-switch trials, participants are required to quickly respond with “go” or “stop” when the experimenter reads the words “green” or “red,” respectively. On Switch trials, participants are required to respond “stop” or “go” when the experimenter reads the words “green” or “red,” respectively. The task includes 18 No-switch and 14 Switch trials, and the score is derived from the total number of correct responses (0–32). *Inductive reasoning* is assessed with the Number Series subscale, in which participants read a brief series of numbers and are instructed to identify the next number in the pattern. The score reflects the total number of correct answers (0–5). *Speed of processing* is assessed with the Backward Counting subscale in which participants verbally count backwards beginning at 100 for 30 seconds. The score reflects the total number of correct numbers listed. The subscales were completed in the following order for all participants: (i) Word List Immediate Recall; (ii) Digit Span Backwards; (iii) Category Fluency; (iv) Stop and Go Switch Task; (v) Number Series; (vi) Backwards Counting; and (vii) Word List Delayed Recall. The task takes approximately 20 minutes to complete.

##### Quantity–Accuracy Profile (QAP)

The QAP (50) is a 60-item multiple-choice, general knowledge questionnaire that measures semantic memory functioning. The updated English version (88) includes questions such as “In biology, what is the process by which carbon dioxide is converted to sugar in plants?” and “What is the capital city of Argentina?”. The task includes a forced-report and a free-report phase. In the forced-report phase, participants are required to select one of five potential answers for each question and rate their confidence in the accuracy of their answer on a scale ranging from 20 to 100%. In the free-report phase, participants are shown the same questions and their corresponding answers, but not their confidence ratings, and are given the choice to report or not report their response. The semantic memory score computed in the current study reflected the free-report accuracy which was the number of correct answers divided by the total number answers reported in the free-report phase.

#### Blood sampling for APOE genotyping

Participants provided dried blood spots which were self-collected under the supervision of a trained research assistant. Briefly, a participant cleaned their hands with soap and water, selected a finger to use for blood spot donation, and wiped the tip of the selected finger with an isopropyl alcohol pad. After a brief period of air drying, the selected finger was warmed for approximately 1 minute. Blood was collected via lancet puncture of the finger pad capillary bed on either side of the center of the selected finger. The first drop of blood was wiped away with sterile gauze and discarded. The next drop of blood (∼30 µL) was deposited directly onto the tip of a Mitra microsampler device (Neoteryx, Torrance, CA, USA) and allowed to air dry completely at room temperature for a minimum of 3 hours. DNA was isolated from the Mitra device using the Maxwell RSC instrument (Promega, Madison, WI, USA) according to the manufacturer's instructions in the customized Product Application Note (RSC FFPE Plus DNA Kit; catalog #AS1720; Application Note “Automated DNA Purification from Blood on a Mitra Microsampler”). Purified DNA was quantitated via Nanodrop (Thermo Fisher Scientific, Waltham, MA, USA), and 18 ng of DNA was used to determine APOE genotypes (at SNPS rs429358 and rs7412) via TaqMan chemistry (Thermo Fisher Scientific) using Fast Advanced Master Mix and assay IDs C___3084793_20 and C____904973_10 according to the manufacturer's suggestions on the QuantStudio 6 Flex instrument (Thermo Fisher Scientific). All experimental samples were genotyped in parallel with sequence-confirmed control samples representing the six common APOE genotypes to aid in cluster anchoring during genotype calling. All genotype calls were derived from the automated calling algorithm in the QuantStudio Real Time PCR Software (Thermo Fisher Scientific).

### Statistical modeling

Statistical analyses were conducted using regression models separately for S-PEST and PHIT. Specifically, for the continuous outcome variable from S-PEST (*d*′ scores), we conducted multiple linear regression models; for the ordinal outcome variable from PHIT (susceptibility score), we conducted ordinal logistic regression models. All regression models included the main effect of chronological age (continuous), and its interaction with APOE4 status (0 = APOE4 noncarriers, 1 = APOE4 carriers) and cognitive functioning scores (continuous) as well as the main effects of each of these moderators. To control for multicollinearity between cognition scores and chronological age, we removed the covariance with age for each of the scores and used the unstandardized residuals as predictors in the regression analyses. Participant sex, years of education, income, marital status, and computer literacy scores were added as covariates in all models. All analyses were conducted using R version 4.1.2 (The R Foundation), and figures were produced using the ggplot2 and sjPlot packages in R.

## Supplementary Material

pgae296_Supplementary_Data

## Data Availability

The full set of data is available on OSF at https://osf.io/dgnjt/?view_only=aab97fa753854a1982f3744a3f268079.

## Notes

a In response to a suggestion from an anonymous reviewer, we re-run the analyses by excluding ε2ε4 allele carriers. Results were largely comparable to those reported in text and reported in Supplemental Information.

b Also see Supplemental Information for results indicating external validity of S-PEST and PHIT regarding real-life fraud victimization.

## Preprints

A preprint for this manuscript is posted on OSF repository https://osf.io/preprints/6f2y9.
