# Supplementary material for: Phishing vulnerability compounded by older age, apolipoprotein E e4 genotype, and lower cognition
Source: PNAS Nexus. 2024 Aug 1;3(8):pgae296. doi: 10.1093/pnasnexus/pgae296 (PMC11309394; doi:10.1093/pnasnexus/pgae296)
Supplement: pgae296_Supplementary_Data [file pgae296_supplementary_data.docx]

**Supplemental Information**

**Phishing Vulnerability Compounded by Older Age, APOE4 Genotype, and Lower Cognition**

Didem Pehlivanoglu, Alayna Shoenfelt, Ziad Hakim, Amber Heemskerk, Jialong Zhen, Mario Mosqueda, Robert C. Wilson, Matthew Huentelman, Matthew D. Grilli, Gary Turner, R. Nathan Spreng, & Natalie C. Ebner

**Corresponding Author:** Didem Pehlivanoglu ([dpehlivanoglu@ufl.edu](mailto:dpehlivanoglu@ufl.edu))

**This PDF file includes:** Supplemental information on study methodology and findings from additional analyses.

**
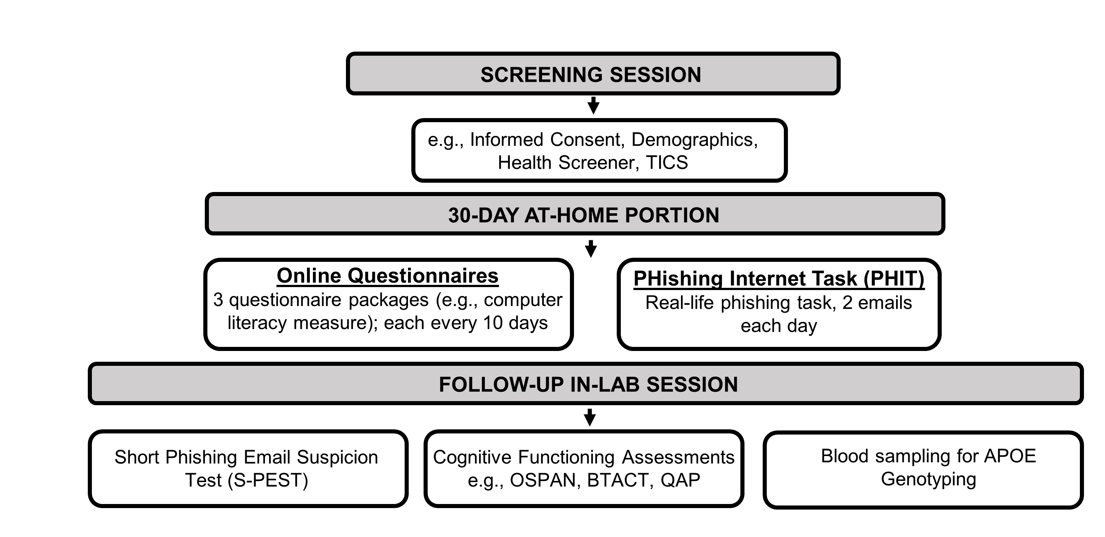
Supplemental Methods**

**Figure S1.** Overview of larger project with measures relevant to this study highlighted. TICS = Telephone Interview for Cognitive Status; OSPAN = Operation Span; BTACT = Brief Test of Adult Cognition by Telephone; QAP = Quantity-Accuracy Profile.

**Supplemental Results**

**Supplemental Result #1:** In response to a suggestion from an anonymous reviewer, we re-run the analyses by excluding ε2ε4 allele carriers (N = 3 participants were removed). Results from this re-analysis were largely comparable to our original findings reported in the main text. In particular, for S-PEST, the interaction between *chronological age*, *APOE4 status*, and *cognitive functioning* remained significant (*B* = 0.007, *t* = 2.10, *p* = 0.037, 95% CI = [0.001, 0.015], indicating that older individuals who were ε3ε4 or ε4ε4 carriers with lower working memory was associated with reduced discrimination ability between phishing and safe emails in the lab. For PHIT, this interaction became marginally significant (*B* = -0.029, *z* = -1.88, *p* = 0.061, 95% CI = [-0.059, 0.001]), indicating a trend that older age in individuals who were carriers of ε3ε4 or ε4ε4 with lower working memory was associated with higher susceptibility to phishing emails in real life. We, however, chose to keep findings from the original analytical approach in the main text based on evidence that ε2 in the presence of ε4 has not been found protective of AD. In particular, a study with more than 5,000 clinically and neuropathologically characterized AD cases demonstrated that while APOE2 homozygotes (ε2ε2) have an exceptionally low likelihood of AD, individuals with the ε2ε4 allele combination have still elevated risk for AD compared to those with the ε3ε3 genotype^1^ (for details see odd ratios reported in Table 1; please also see Golberg et al. and Oveisgharan et al. for similar results showing that the ε2ε4 genotype was associated with increased risk of AD pathology^2,3^ as well as a meta-analysis by Farrer et al. showing increased odds of AD among ε2ε4 carriers^4^).

**Supplemental Result #2:** In response to a question from an anonymous reviewer, Table S1 presents the Age Group x Sex x Race breakdown along with results from corresponding chi-square tests. In particular, we first binned the chronological age variable into 3 categories to reflect young, middle-aged, and older adult age categories. As can be seen in the table, participants were predominantly female, with comparable sex distributions across the three age groups (*χ*^2^ = 2.93, *p* = .231). The distribution of race, however, significantly differed between the age groups (*χ*^2^ = 41.83, *p* < .001), with older adults being predominantly White and less diverse than young and middle-aged adults. We re-ran the analyses reported in the main text by controlling for race; all results remained the same.

Table S1. *Sex and Race Distributions by Age Groups*

| **Age Group** | **Young**  18-34 yrs.  *N* = 73 | **Middle-aged**  35-64 yrs.  *N* = 54 | **Older**  65-90 yrs.  *N* = 55 |
| --- | --- | --- | --- |
|  | ***%*** | ***%*** | ***% χ^2^  p*** |
| **Sex** |  |  | **2.93 .231** |
| Female | 84 | 78 | 71 |
| Male | 16 | 22 | 29 |
| **Race** |  |  | **41.83 <.001** |
| White | 64 | 72 | 96 |
| Black/African | 6 | 20 | 0 |
| Asian | 19 | 0 | 0 |
| Other | 11 | 8 | 4 |

**Supplemental Result #3:** In response to a comment from an anonymous reviewer regarding external validity of S-PEST and PHIT, we leveraged two self-report items about history of financial fraud participants has experienced in their real life, that were asked as part of our exit survey and phrased as follows:

**Q1)** Have you ever been the victim of a scam? Yes No

**Q2)** If you realized that you were getting scammed, were you able to stop the scam? Yes No

We conducted two separate logistic regression models on participant responses to these two items, respectively, with S-PEST and PHIT scores as predictors. We found that participants who were more susceptible to our email phishing in PHIT had become victim of a scam in their real life (assessed via Q1; *z* = 2.21, *p* = .027); there was no significant effect of S-PEST performance on this item (*z* = 0.13, *p* = .894). In addition, participants who had lower discrimination ability between phishing and safe emails in S-PEST had not been able to stop a scam in their real life (assessed via Q2; *z* = 2.04, *p* = .042); there was no significant effect of PHIT performance on this item (*z* = -0.61, *p* = .539). These findings further support the external validity of our in-lab controlled (S-PEST) and simulated (PHIT) phishing susceptibility measures. We, however, would like to highlight that these findings are somewhat preliminary in nature and should be interpreted with caution given that only 26.5% (N = 48) of participants indicated previous scam victimization (in response to Q1) and only 4.3% (N = 8) of participants indicated that they had not been able to stop the scheme (in response to Q2). These numbers are in line with statistics of fraud going underreported, especially among older adults^5^, possibly due to embarrassment and/or fear of reporting or due to lack of awareness of the victimization^6^, severely underestimating the real magnitude of the problem^7^.

**References**

1. Reiman, E. M. *et al.* Exceptionally low likelihood of Alzheimer’s dementia in APOE2 homozygotes from a 5,000-person neuropathological study. *Nat Commun* **11**, 667 (2020).

2. Goldberg, T. E., Huey, E. D. & Devanand, D. P. Association of APOE e2 genotype with Alzheimer’s and non-Alzheimer’s neurodegenerative pathologies. *Nat Commun* **11**, 4727 (2020).

3. Oveisgharan, S. *et al.* APOE ε2ε4 genotype, incident AD and MCI, cognitive decline, and AD pathology in older adults. *Neurology* **90**, e2127–e2134 (2018).

4. Farrer, L. A. *et al.* Effects of Age, Sex, and Ethnicity on the Association Between Apolipoprotein E Genotype and Alzheimer Disease: A Meta-analysis. *JAMA* **278**, 1349–1356 (1997).

5. Pak, K., Shadel, D. & Office, A. W. S. AARP Foundation National Fraud Victim Study.

6. Button, M., Lewis, C. & Tapley, J. Not a victimless crime: The impact of fraud on individual victims and their families. *Secur J* **27**, 36–54 (2014).

7. Beals, M. E., Carr, D. C., Mottola, G. R., Deevy, M. J. & Carstensen, L. L. How Does Survey Context Impact Self-reported Fraud Victimization? *The Gerontologist* **57**, 329–340 (2017).
